# Alterations of lung microbiota in patients with non-small cell lung cancer

**DOI:** 10.1080/21655979.2022.2045843

**Published:** 2022-03-07

**Authors:** Wen Zeng, ChengZhu Zhao, Mengge Yu, Hailong Chen, Yiyun Pan, Yuhuan Wang, Hejing Bao, Hao Ma, Shudong Ma

**Affiliations:** a Department of Oncology, Nanfang Hospital, Southern Medical University, Guangzhou, Guangdong, China; b Department of Oncology, Ganzhou Cancer Hospital, Gannan Medical University,Ganzhou, Jiangxi, China

**Keywords:** Non-small cell lung cancer, microbiota, microbial diversity, 16S rRNA gene, veillonella

## Abstract

The role of lung microbiota in non-small cell lung cancer remains unclear. We investigated the characteristics and functional roles of lung microbiota in non-small cell lung cancer. Bronchoalveolar lavage fluid samples were obtained from patients with non-small cell lung cancer (n = 46) and with benign lung disease (n = 29). The differences in composition and gene expression in the microbiota between the samples were analyzed using 16s rRNA sequencing. The oncogenic genus (*Veillonella*) was then evaluated in the progression of lung cancer in C57 BL/6 mice. Compared to benign lung disease, the lung microbiota in non-small cell lung cancer was significantly altered, both in terms of α- and β-diversity. In terms of bacterial composition, the non-small cell lung cancer group was enriched with two Phyla (Firmicutes, Bacteroidetes) and three genera (*Streptococcus, Prevotella, Veillonella*). *Prevotella* and *Veillonella* were most strongly associated with non-small cell lung cancer, and *Veillonella* significantly promoted the progression of lung cancer *in vivo*. Moreover, metabolic prediction revealed that ribosomes, biosynthesis of secondary metabolites, and pyrimidine metabolism were among the enriched pathways that may be involved in the progression of non-small cell lung cancer. Overall, results suggest that the progression of non-small cell lung cancer is followed by significant changes in the composition and function of the lung microbiota. These differing genera may be potential diagnostic markers and therapeutic targets.

## Graphical abstract

**Figure:**
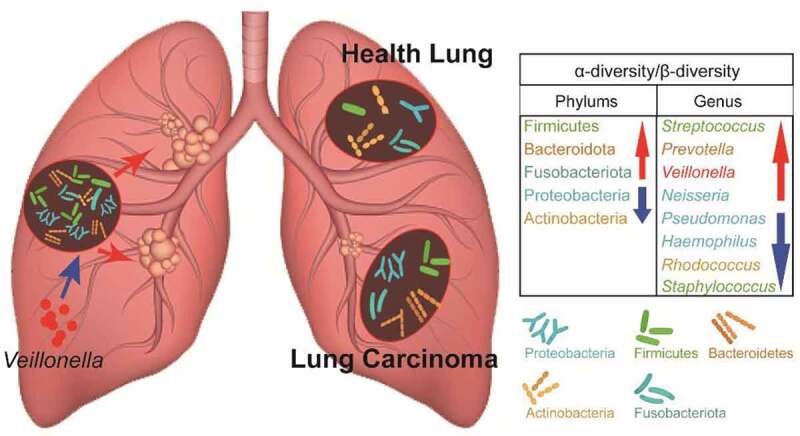

## 1. Introduction

Lung cancer is one of the most common cancers and causes of cancer-related deaths globally. Each year, over 1.8 million people are diagnosed with lung cancer, and 1.6 million of them succumb to the disease. Non-small cell lung cancer (NSCLC) is the most common type of lung cancer pathology and is caused by environmental factors and host genetics [1]. Smoking is also recognized as a major known risk factor for lung cancer, but less than 15% of patients with NSCLC smoke [2]. Chronic obstructive pneumonia, bronchitis, and pneumonia are associated with an increased risk of lung cancer [3], and inflammatory diseases have overlapping pathways with those implicated in the pathogenesis of NSCLC [4]. Furthermore, although the genomic profile of NSCLC has been extensively described, the external factors that influence the development of NSCLC remain unclear [5].

Since studies have reported a causal relationship between *Helicobacter pylori* and gastric cancer [6], there is growing evidence that local microorganisms play a key role in cancer pathogenesis and treatment, such as *Fusobacterium nucleatum* promoting colon cancer and *Enterotoxigenic Bacteroides fragilis* promoting breast cancer [7,8]. The lung microbiota is involved in the regulation of the host pulmonary immune system, and the balance between pulmonary immunity and microbiota is crucial for protection against infection [9]. Dysbiosis of the lung microbiota can induce activation of resident immune cells, such as M1 macrophages and γδ T cells, and induce oxygen free radicals and gene mutations, thus promoting lung carcinogenesis [10]. In mouse models, dysregulation of lung bacteria generates a pro-inflammatory environment and promotes tumor growth. However, the relationship between the function of the lung microbiota and the host in the context of lung cancer remains unknown.

In this study, we hypothesized that there exists a significant change in composition and function of the lung microbiota during the progression of NSCLC. Newly diagnosed NSCLC patients are suitable for profiling the relationship between lung microbiota and cancers. To test this hypothesis, we examined the microbiota composition of bronchoalveolar lavage fluid (BALF) from 46 patients with newly diagnosed NSCLC and 29 patients with benign lung disease using 16s rRNA sequencing and searched for associations between NSCLC and the microbiota through bioinformatic analysis and *in vivo* experiments. We aim for our results to provide a reference for the clinical application of lung microbiota evaluation in the diagnosis and treatment of NSCLC.

## 2. Materials and methods

### 2.1 Patients and samples collection

A total of 75 patients who underwent computed tomography examination suggestive of suspicious lung nodules and clinical bronchoscopy at the Ganzhou cancer hospital (Jiangxi, China) from 1 December 2020 to November 31, 2021, were enrolled in this study. The patients included those with severe bronchitis, chronic obstructive pneumonia, bronchiectasis, and pulmonary fibrosis. Forty-six patients diagnosed with NSCLC were included in the newly diagnosed lung cancer group, and 29 patients with a pathological diagnosis of benign lesions were included in the control group. No antibiotics were administered within 4 weeks of enrollment. Population and clinical data were recorded, including age, sex, body mass index (BMI), smoking history, type of pathology, and tumor stage. The study was approved by the Ethics Committee of Ganzhou cancer hospital (2021 Research Ethics Review No. 5), and all enrolled patients signed an informed consent form.

BALF was collected by an experienced clinician following the optical fiber bronchoscopy protocol to avoid oral contamination to the greatest extent possible. In total, 10–15 mL of BALF were collected from each patient, filtered through a single layer of gauze to remove mucus, and centrifuged at 10,000 *g* for 10 min. The precipitate was then collected and frozen at −80°C for further use.

### 2.2 DNA extraction and polymerase chain reaction amplification

Total genomic DNA was extracted from BALF using a QIAamp DNA Mini Kit (Qiagen, Hilden, Germany). DNA purity and concentration were measured using a NanoDrop 2000 (Thermo Scientific, USA), and the integrity of the extracted DNA was determined by 1% agarose gel electrophoresis. Polymerase chain reaction (PCR) amplification was performed using TransStart Fastpfu DNA Polymerase (AP221-02, TransGen, Beijing, China), using the following primers to amplify the 16s rRNA ‘V3-V4’ sequence: 338 F ‘ACTCCTACGGGAGGCAGCAG’ and 806 R ‘GGACTACHVGGGTWTCTAAT’. The V3-V4 region sequences were sequenced and analyzed.

### 2.3 Gene sequencing

The 16s rRNA V3-V4 region genes in the samples were sequenced in both directions using the Illumina MiSeq high-throughput platform (Meiji Biological Co., Ltd. Shanghai, China). The paired-end reads obtained from MiSeq sequencing were first spliced based on overlap relationships, while the sequence quality was quality controlled and filtered, then analyzed using QIIME2, and the reads were clustered into operational taxonomic units (OTUs) using UCLUST with 97% similarity [11]. Taxonomic analysis of OTUs was performed according to Greengene (Release 13.5 http://greengenes.secondgenome.com/) [12].

### 2.4 Sequencing analysis

Shannon index, Simpson diversity index, Sobs index, and Chao1 index diversity were used for alpha (α)-diversity analysis of species, and the Wilcoxon rank-sum test was used to test for differences between index groups. Similarities or differences in community composition between different grouped samples underwent principle coordinate analysis (PCoA), analysis of similarities (ANOSIM), partial least squares discriminant analysis (PLS-DA) based on the principal coordinates of Bray Curtis, and analysis of unweighted and weighted UniFrac distance matrices [13]. The linear discriminant analysis (LDA) effect size (LEfSe) algorithm (huttenhower.sph.harvard.edu/galaxy/) was used for the non-parametric Kruskal–Wallis sum-rank test to detect differences in species abundance between groups and to obtain significantly different species and metabolic pathways [14]. LDA was used to evaluate the magnitude of the effect of different species or metabolic pathways on the difference between groups, with taxa with an LDA score >4 and a p-value <0.05 being considered significantly enriched. Species correlation networks were constructed from one-way network analysis to analyze species interactions in the environment, and correlation coefficients were described using a heat map. Reconstruction of Unobserved States2 (PICRUSt2) was used to predict the functional composition of the taxa in the samples from amplicon sequencing results, and the predicted Kyoto Encyclopedia of Genes and Genomes (KEGG) pathway level 3 was analyzed descriptively.

### 2.5 Bacterial strains, cell line, and culture conditions

*Veillonella parvula* ATCC 10790 (0867, ATCC, USA) was purchased from ATCC and maintained in Wilkins-Chalgren anaerobe broth (Thermo Fisher Scientific, USA) in an anaerobic jar (80% N2, 10% H2, 10% CO_2_) at 37°C. The Lewis lung cancer (LLC) cell line was purchased from Procell Life Science & Technology Co., Ltd (Wuhan, China). Cells were maintained in Dulbecco’s Modified Eagle Medium (Gibico, USA) supplemented with 10% fetal bovine serum (Gibico, USA).

### 2.6 Syngeneic tumor models

Six- to 8-week-old C57 bl/6 j male mice were obtained from Southern Medical University (Guangzhou, China). LLC cells (5 × 10^5^/100 μl) were injected either subcutaneously on the left flank region or intratracheal instillation, and the animals were kept in a specific pathogen-free environment. Mice were executed at the fourth week after inoculation with LLC cells, and tumor weights and volumes were recorded. Volume (mm^3^) was calculated as L*W*W/2, with L for the length of the tumor and W for the width of the tumor (in centimeters).

### 2.7 Statistical analysis.

The student’s t-test was performed to compare the variables between the two sample groups. Data are expressed as the mean ± standard deviation or mean ± standard error of the mean, as indicated in the figure legends. Statistical tests were two-tailed, and statistical significance was set at P < 0.05.

## 3. Results

### 3.1 Overview

We conducted a cross-sectional study to compare the differences in composition between the lung microbiota of NSCLC and benign lung disease by 16S rRNA gene sequencing. The diversity and composition of the lung microbiota differed significantly between the NSCLC group and the control group. The lung microbiota influenced NSCLC through metabolic pathways, and the *Veillonella* genus was identified as having an oncogenic role in promoting lung adenocarcinoma progression in mice.

### 3.2 Characteristics of the participants

To assess the variation and role of lower respiratory microbiota in lung cancer, the composition of BALF microbiota was examined using 16s rRNA gene sequencing in 29 control subjects and 46 patients with lung cancer. The median age was 63.8 ± 11.2 in the lung cancer group and 64.2 ± 8.4 in the control group. These patients did not have a serious infection, nor were they treated with antibiotics in the last 4 weeks before enrollment. Patients in both groups had similar age, BMI, male/female ratio, and comparable baseline information. Patients in the lung cancer group were all diagnosed with NSCLC (25 with adenocarcinomas and 21 with squamous carcinomas), and the control group was dominated by benign lung nodules (inflammatory pseudotumors, n = 23; malignant hemangiomas, n = 4; and sclerosing hemangiomas, n = 2). The stage of patients in the lung cancer group was predominantly intermediate and advanced, including 3 patients with stage I, 7 with stage II, 22 with stage III, 14 with stage IV, 13 with distant metastasis, and 1 with non-metastasis. Overall, the trends observed in the enrolled patients were consistent with the epidemiological trends of lung cancer (Table 1).

**Table 1.** Baseline characteristics of the patients

	Non-small cell Lung cancer(n = 46)	Control(n = 29)	P value*
**Demographics/anthropometric**			
Age/year	63.8 ± 11.2	64.2 ± 8.4	0.8693
Male/Female	34/12	21/8	0.8871
BMI(kg/m2)	23.24 ± 2.12	23.54 ± 2.32	0.5668
**Tumor stage (%)**			
I	3	N/A	
II	7	N/A	
III	22	N/A	
IV	14	N/A	
**Tumor Type(%)**			
ADC	25	N/A	
LCC	21	N/A	
**Tumor metastasis**			
Metastasis	13	N/A	
Non-metastasis	1	N/A	
**Smoking status (%)**			
Never smoker	9	7	
Ever smoker	37	22	

**N/A: Not applicable**

### 3.3 Lung microbiome profiles analyzed by 16s rRNA sequencing

**Figure 1.**
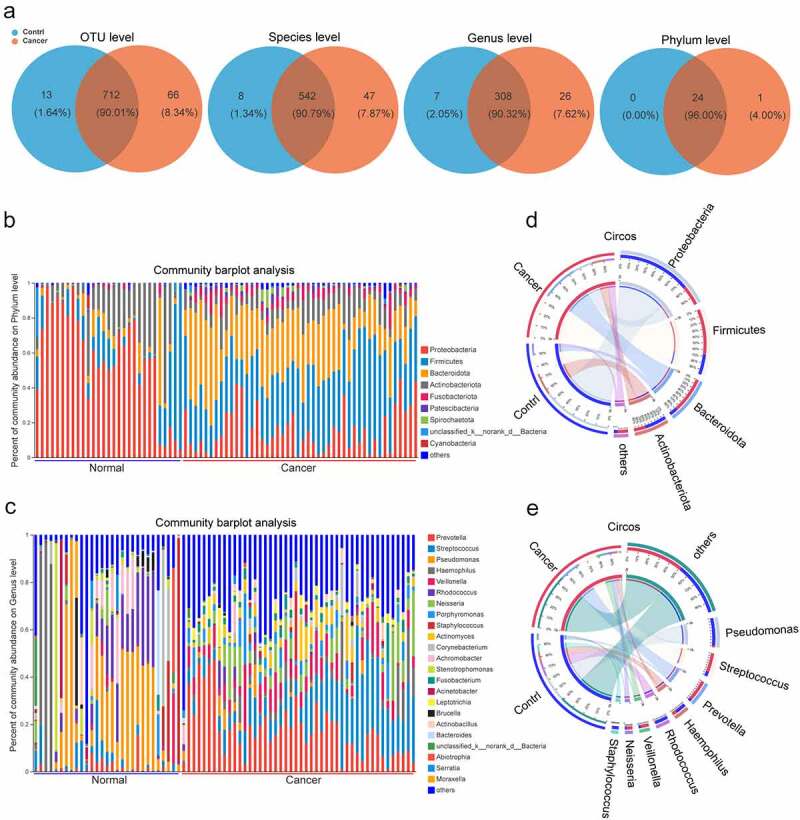
The profiles of lung microbiome composition in the control and lung cancer groups. (a). Venn diagrams show the numbers of microbiota at OTUs, species, genera, phyla commonly shared between both control and lung cancer groups. (b, c) Heatmaps show the relative frequency of lung microbiota at the phylum (b) and genus levels (c) in each sample. (d, e). Circos show the composition of lung microbiota between the control and lung cancer groups at the phylum (d) and genera (e) levels. The red and blue dots represent the cancer and control samples, respectively.

During the analysis of 16s rRNA sequencing data from BALF from 75 samples, we screened 4,272,518 sequences with an average length of 421, which were then annotated in the rRNA library database (Greengenes) and analyzed for OTUs (Table S1). Taxonomic analysis of OTU representative sequences at 97% similarity level, sequence sampling, and exclusion of species with sequence sum <100 resulted in a total of 791 OTUs. Twenty-four and 25 OTUs were identified in the control and lung cancer groups, respectively, at the phylum, class, order, family, genus, and species levels. A detailed analysis of the unique microbial taxa in the different groups including a total of 66 OTUs, 47 species, 26 genera, and 1 phylum were detectable only in the cancer group, whereas another 13 OTUs, 8 species, and 7 genera were only detectable in the control group (Figure 1a, Table S2).

We found that the process of lung carcinogenesis was accompanied by significant changes in the composition of the lower respiratory microbiota. At the phylum level, the top five in abundance were *Proteobacteria, Firmicutes, Bacteroidetes*, Actinobacteria, and Fusobacteriota. Proteobacteria and Actinobacteria showed high abundance in the control group, while Firmicutes, Bacteroidota, and Fusobacteriota were highly abundant in the lung cancer group (Figure 1b, d). At the genus level, the top 10 genera, *Prevotella, Streptococcus, Pseudomonas, Haemophilus, Veillonella, Rhodococcus, Neisseria, Porphyromonas, Staphylococcus*, and *Actinomyces*, were most common between the control and lung cancer groups, with *Pseudomonas, Haemophilus, Rhodococcus*, and *Staphylococcus* being highly abundant in the control group, while *Streptococcus, Prevotella, Veillonella*, and *Neisseria* were highly abundant in the lung cancer group (Figure 1c, e).

### 3.4 Biodiversity in the lung microbiota in NSCLC

**Figure 2.**
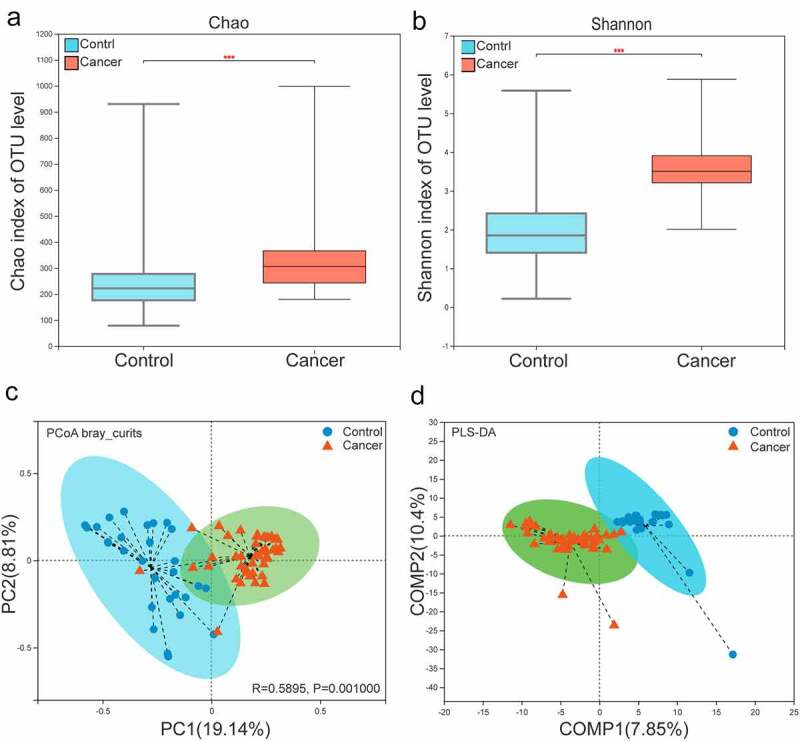
Analysis of α- and β-diversity in the microbial community of BALF samples from the NSCLC and control group. (a, b) Parameters Chao1 (a) and Shannon (b) indices were used for α-diversity analysis. (c) PCoA plots of Bray Curtis distance matrix. (d). Partial least squares discriminant analysis (PLS-DA) of β-diversity. The red and blue dots represent the cancer and control samples, respectively.

The α-diversity in the bacterial community in the lower respiratory tract differed between patients in the lung cancer group and patients in the control group. The Sobs and Chao indices reflected the difference in community richness between the controls and NSCLC patients(p < 0.001).Shannon and Simpson’s indices reflected the difference in two groups(p < 0.001). These results showed lower respiratory microbiota may change considerably during lung carcinogenesis(Figure 2a, B, Figure S1A, B).

The composition of the lower respiratory microbiota of lung cancer samples and control samples was further assessed for a clear trend of segregation based on analysis of OTU levels. Various β diversity indices(PCoA analysis of Bray Curtis, weighted UniFrac and unweighted UniFrac analysis) revealed he bacterial community composition was determined to be significantly different between the NSCLC and control samples (p = 0.001) (Figure 2c, Figure S1C, D). In addition, PLS-DA also showed that the bacterial community composition was significantly different between two groups (Figure 2d). Therefore, the composition of the lower respiratory microbiota was significantly alter in progression of NSCLC.

### 3.5 Distinct taxa in BALF samples between NSCLC and controls

**Figure 3.**
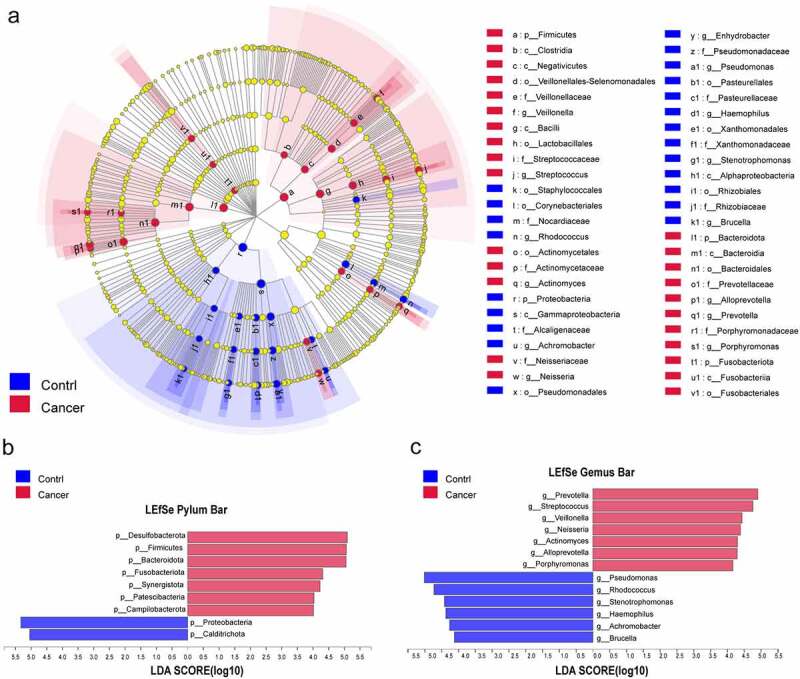
Distinct taxa were characterized in BALF samples by applying LEfSe analysis. (a) A Cladogram was constructed using the LEfSe method to represent the phylogenetic distribution of bacteria. (b,c) Linear discriminant analysis (LDA) scores show bacterial within two groups at the phylum level (b) and genus level (c). Blue color represents taxa enriched in the control patients, and red color represents taxa enriched in NSCLC patients.

The LEfSe method was implemented, and this analysis (LDA>4) showed significant differences in the microbiota of BALF samples between the control and lung cancer groups (Figure 3a). Compared to the control group, Desulfobacterota, Firmicutes, Bacteroidetes, Fusobacteriota, Synergistota, Patescibacteria, and Campilobacterota were significantly enriched in the NSCLC group at the phylum level, while Proteobacteria and Calditrichota were enriched in the control samples (Figure 3b). At the genus level, *Prevotella, Streptococcus, Veillonella, Neisseria, Actinomyces, Alloprevotella*, and *Porphyromonas* were enriched in the NSCLC samples, while *Pseudomonas, Rhodococcus, Stenotrophomonas, Haemophilus, Achromobacter*, and *Brucella* were enriched in the control samples (Figure 3c). In addition, we also analyzed the differences in lung microbiota at the class, family, and species levels, contributing to a broader understanding of the important microbiota that distinguishes NSCLC from normal controls (Figure S2A-C).

### 3.6 Co-occurrence network analysis of microbiota in NSCLC

**Figure 4.**
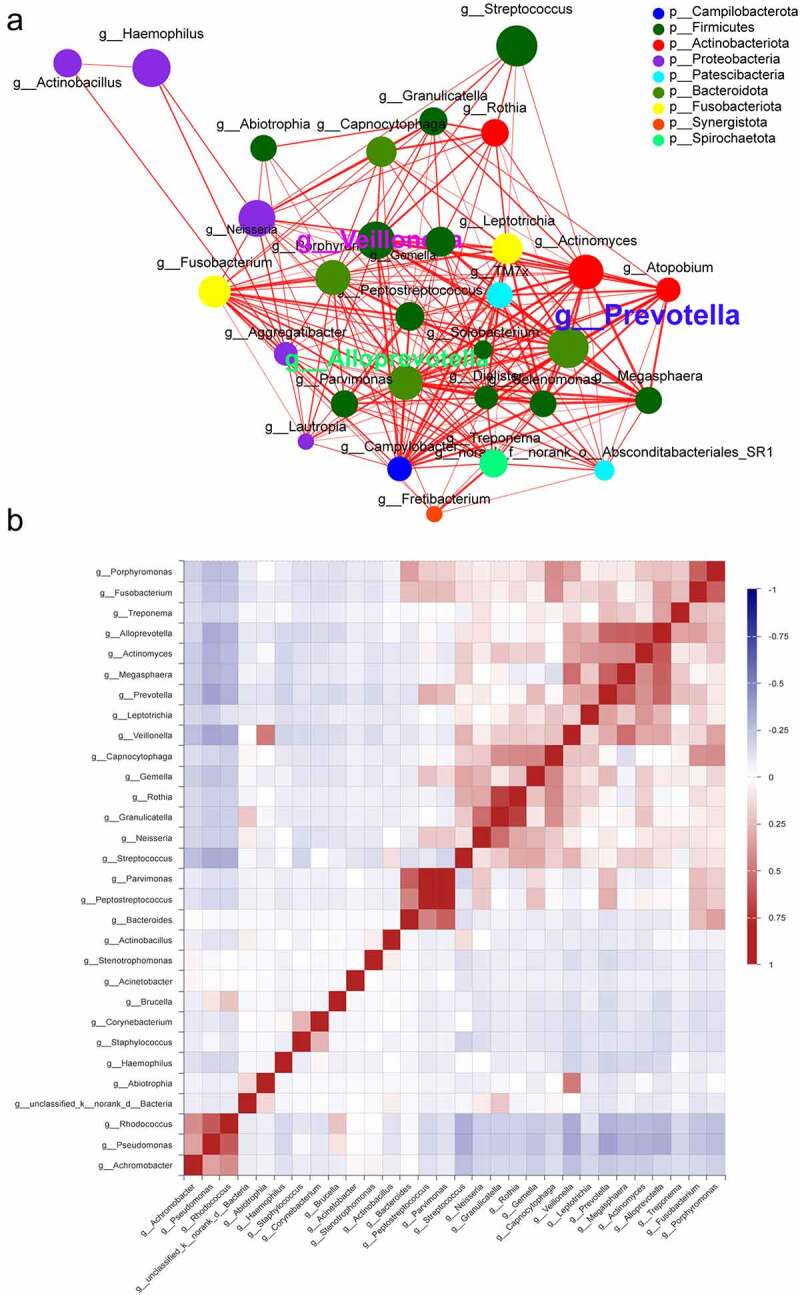
Co-occurrence network of the microbiome at the genus level. (a) Each node represents a genus, colored according to its phylum level, and each edge indicates an important symbiosis, which is colored according to its relevance (red: positive, green: negative). (b) Pearson correlations were calculated and analyzed between the top 50 most abundant bacterial genus. Correlation values range from −1.00 (blue) to 1.00 (red).

The genus-level correlation network plots revealed significant interactions between different genera, with *Prevotella, Alloprevotella, Veillonella, Pseudomonas*, and *Rhodococcus* being the five most significantly associated genera in the network. The first three genera were enriched in the lung cancer group and the latter two were enriched in the control group (Figure 4a). In the lung cancer group, *Prevotella* and *Prevotella* were positively correlated with Megasphaera (ρ = 0.57595, ρ = 0.5275), and *Alloprevotella* was positively correlated with *Actinomyces* (ρ = 0.60058), whereas in the control group, *Pseudomonas* was positively correlated with *Rhodococcus* (ρ = 0.59812). Genus-to-genus and genus-to-sample interactions are important factors influencing lung cancer progression (Figure 4b).

### 3.7 Analysis of metabolic pathways of lung microbiota in NSCLC

**Figure 5.**
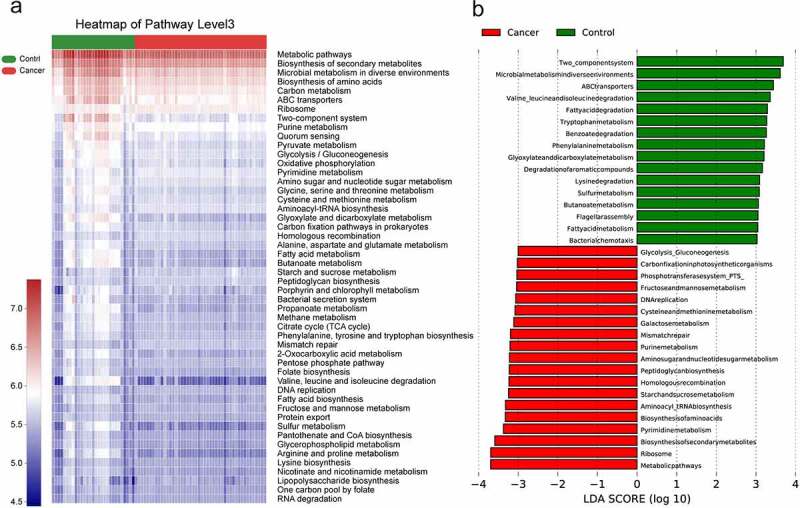
Predicting gene function in the lung microbiota using PICRUSt2.The impact of differentially enriched KEGG pathways between lung cancer and the control group is shown using a heat map (a) and evaluated through the LDA score. Only KEGG pathways meeting an LDA score >3.0 are shown (b). The columns in red and blue represent the cancer and control groups, respectively.

The differential functions of bacterial communities were analyzed using by PICRUSt2, followed by LEfSe analysis KEGG functional predictions in cellular processes, human diseases, metabolism, environmental information processing, genetic information processing, and organismal systems. The differences between the NSCLC and control groups are shown in Figure 5a. Overall, 16 and 20 different pathways were enriched in the control and NSCLC groups, respectively, with metabolic pathways, ribosome, biosynthesis of secondary metabolites, and pyrimidine metabolism being the distinct metabolic processes identified in the lung cancer group, while two-component system, microbial metabolism in diverse environments, and ABC transporters were significantly present in the control group.

Further classification of KEGG functional predictions into cellular processes, human diseases, metabolism, organismal systems, and environmental information processing suggested that the different metabolic functions of the microbiota may be an important factor influencing the progression of lung cancer (see Table 1) (Figure S3).

### 3.8 Veillonella *promotes the growth of LLC tumors* in vivo

**Figure 6.**
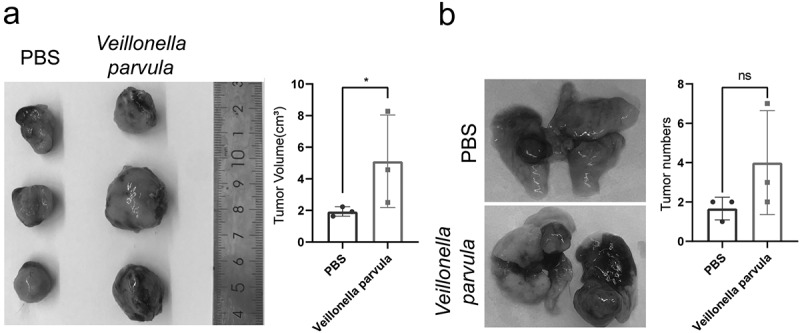
*Veillonella* promotes the growth of Lewis lung cancer (LLC) tumors *in vivo*. (a) C57BL/6 mice after subcutaneous transplantation with LLC cells, *Veillonella parvula* injections around the tumor twice a week after tumor cell engraftment; PBS was used as control. Data were analyzed using student’s test (n = 3 per group). (b) C57BL/6 mice after orthotopic transplantation with LLC cells, and intratracheal infusion of *Veillonella parvula* twice a week after tumor cell engraftment; PBS was used as control. Data were analyzed using student’s test (n = 3 per group). *P < 0.05; ns, not significant.

To further observe the effect of the key genus in the lung on the growth of NSCLC, we used C57BL/6 mice and LLC cell lines for subcutaneous transplantation of tumors and in situ cancer models to conduct *in vivo* studies on the interaction of bacteria with lung cancer. Peri-tumor injection with *Veillonella parvula* in LLC subcutaneous tumors significantly promoted the growth of LLC transplanted tumors in a relatively short period (Figure 6a). In contrast, intratracheal instillation of *Veillonella parvula* in mice with LLC in situ tumors revealed different dynamics in tumor growth. Specifically, *Veillonella parvula* instillation caused disruption of the lung microbiota and did not affect the growth of individual tumors (Figure 6b), but this difference was not statistically significant.

## 4. Discussion

The main causative factors of lung cancer are exposure to carcinogenic substances such as cigarette smoke, chronic airway inflammation, lung fibrosis, etc [15,16]. These pathogenic factors may alter the lung microbiota and are associated with lung cancer progression, phenotype, and severity [17,18]. However, little research has been conducted on the direct relationship between lung cancer and the lung microbiota. Bronchoalveolar lavage, bronchial brushing tissue, buccal samples, surgical resection tissue, or exhaled breath condensate are used for the common specimens characterizing the lung microbiota, and although the microbiota of the lower and upper respiratory tracts are highly similar [19,20], the dysregulated characteristics of the lower respiratory microbiota are more suitable for distinguishing lung cancer from benign lung disease [21].

Comparing the microbiota of BALF samples between patients with NSCLC and control patients, the lung microbiota of NSCLC showed a more complex diversity with higher abundance and α-diversity. Laroumagne et al. [22] showed that the abundance and diversity of the microbiota in the lower lung in patients with lung cancer were significantly reduced compared to healthy individuals. Peters et al. showed that the α-diversity of the microbiota was higher in primary or recurrent lung cancer than in normal lung tissue [23,24]. In contrast, Sang [25] found no difference in α- and β-diversity between the two groups. This difference in findings may be due to differences related to geographical differences, type, and the number of samples collected, as well as the analysis of sequencing data. In the current study, ANOSIM further confirmed the significant differences in the microbiota between lung cancer lesions and controls. Thus, dysregulation in the lung microbiota occurs during NSCLC development.

Among the lung microbiota, Bacteroidetes, Firmicutes, Proteobacteria, and Actinobacteria were the four most common core phyla, while *Prevotella, Streptococcus, Veilonella, Neisseria, Haemophilus*, and *Fusobacterium* were the most abundant genera [26,27]. In a study on postoperative lung tissue microbiota characterization, lung cancer tissue lower airway brush samples (n = 39) showed a stronger enrichment in *Veillonella* and *Streptococcus* than did the benign disease (n = 36) and healthy control samples (n = 10) [28]. In a Korean study, the abundance of *Veillonella* and *Megasphaera* was greater in BALF samples from patients with lung cancer than in those with benign lung lesions (n = 8) [25]. Similar to the previous study, in our study, the lung microbiota in the NSCLC group was enriched at the phylum level for Firmicutes and Bacteroidetes, and the top three enriched genera included *Streptococcus, Prevotella*, and *Veillonella*. Furthermore, in the NSCLC group samples compared to those of the control group, *Prevotella* abundance was increased from 1.93% to 17.73%, that of *Veillonella* from 1.76% to 7.62%, and that of *Streptococcus* from 4.01% to 15.76%. The conclusions differ from the Markus Hilty study, in which *Veillonella* and *Prevotella* were expressed in higher abundance in healthy lungs controls in those with non-lung cancer disease [29]. Although some differences exist, these studies suggest that changes in the dynamics of the bacterial phyla Firmicutes and Bacteroidetes and the genera *Streptococcus, Veillonella*, and *Prevotella* are closely associated with the development of diseases, including lung cancer.

LEfSe analysis was applied to identify species that were different between groups, and LDA was further used to evaluate the effect of these differences in species on disease progression. Previous studies have also been controversial, showing that Firmicutes was enriched and Proteobacteria reduced in lung cancer tissues compared to emphysema samples [30], whereas Proteobacteria enrichment was present in most lung tumors in smokers [31]. Unlike studies on the relationship between Clostridium perfringens and colon cancer, there is a lack of studies on the influence of a single genus of bacteria on lung cancer progression. Tsay et al [21] administered commensal bacteria (*Veillonella*) in a KP mouse model and induced upregulation of Erk/MAPK and inflammatory signaling pathways in respiratory epithelial cells and an increase in Th17 cells through the constructed dysbiosis environment, which in turn [10] significantly promoted the progression of lung cancer when bacteria (*Herbaspirillum* and *Sphingomonadaceae*) isolated from advanced tumors in SPF mice were re-transplanted back into the intestine.

*Prevotella* is an important pathogen of periodontitis and secretes peptides through proteinase-activated receptors (PARs), which are involved in the regulation of proliferation, apoptosis, immunity, cytokine production, and microenvironmental inflammation in oral squamous cell carcinoma [32,33]. Gram-positive (*Streptococcus*) and gram-negative (*Escherichia coli*) bacteria can promote distant metastasis of NSCLC through activation of Toll-like receptors 2/4 and interleukin-6 secretion [34,35]. *Veillonella parvula*, the most commonly known species of the *Veillonella* genus, was found to significantly promote the progression of LLC in mice, consistent with *Veillonella parvula* promoting the formation of lung adenocarcinoma in KPA mice. Thus, this suggests the role and potential mechanisms by which changes in the abundance of core genera affect lung cancer progression. In addition, the role of a smaller proportion of groups in lung cancer should not be ignored as well; further research is needed to explore more causative organisms and their mechanisms of action.

Bacteria coexist in complex networks of interactions, and interactions within these networks affect the species involved, which in turn leads to disease. As shown in our co-expression network relationship diagram, the microbiota in NSCLC exhibit complex ecological relationships, with *Prevotella, Alloprevotella*, and *Veillonella* clustering together to form the densest network of interactions. Similar results to those of LEfSe analysis of key genera further suggest a key role for *Prevotella* and *Veillonella* in the progression of NSCLC, which could distinguish lung cancer from normal samples. Based on 16s rRNA sequencing information, the PICRUSt 2 method was used to make inferential predictions about the function of the bacterial community, focusing mostly on metabolic pathways, ribosome, biosynthesis of secondary metabolites, and microbial metabolism in LEfSe’s analysis of certain gene functions in the community; among the metabolic pathways enriched in NSCLC, gluconeogenesis, amino acid metabolism, and inflammatory signaling pathways were similarly enriched in chronic obstructive pulmonary disease, a risk factor for lung carcinogenesis [36]. In contrast, cell growth, death, reduced transport, and catabolism are associated with reduced transport and catabolic pathways of bacterial carbohydrate metabolism [37]. If more detailed information on the functional changes of the bacteriophage genes is needed, macrogenome sequencing should be performed for further analysis.

## Limitations

This research is a cross-sectional study and does not establish a causal relationship between microbiota and lung cancer, which must be further confirmed by more mechanistic studies on animal and cellular aspects. Furthermore, we hypothesize that *Prevotella* and *Veillonella* are closely associated with NSCLC, but their relevance to clinical and prognostic aspects requires more clinical samples and information for analysis.

## Conclusion

Significant changes in bacterial composition and bacterial gene function were found in NSCLC. In particular, Firmicutes, Bacteroidetes, Fusobacteriota at the phylum level, and *Prevotella, Alloprevotella*, and *Veillonella* at the genus level were significantly enriched in NSCLC samples. We also confirmed that *Veillonella (Parvula)* plays the role of a carcinogenic bacteria for lung cancer *in vivo*. These important genera may be potential diagnostic markers and therapeutic targets.

## Supplementary Material

Supplemental Material

## Data Availability

Data will be provided on reasonable request
