# Supplementary material for: Alterations of lung microbiota in patients with non-small cell lung cancer
Source: Bioengineered. 2022 Mar 7;13(3):6665–77. doi: 10.1080/21655979.2022.2045843 (PMC8973753; doi:10.1080/21655979.2022.2045843)
Supplement: Supplemental Material [file KBIE_A_2045843_SM6988.zip › 补充结果/Figure S-legends.docx]

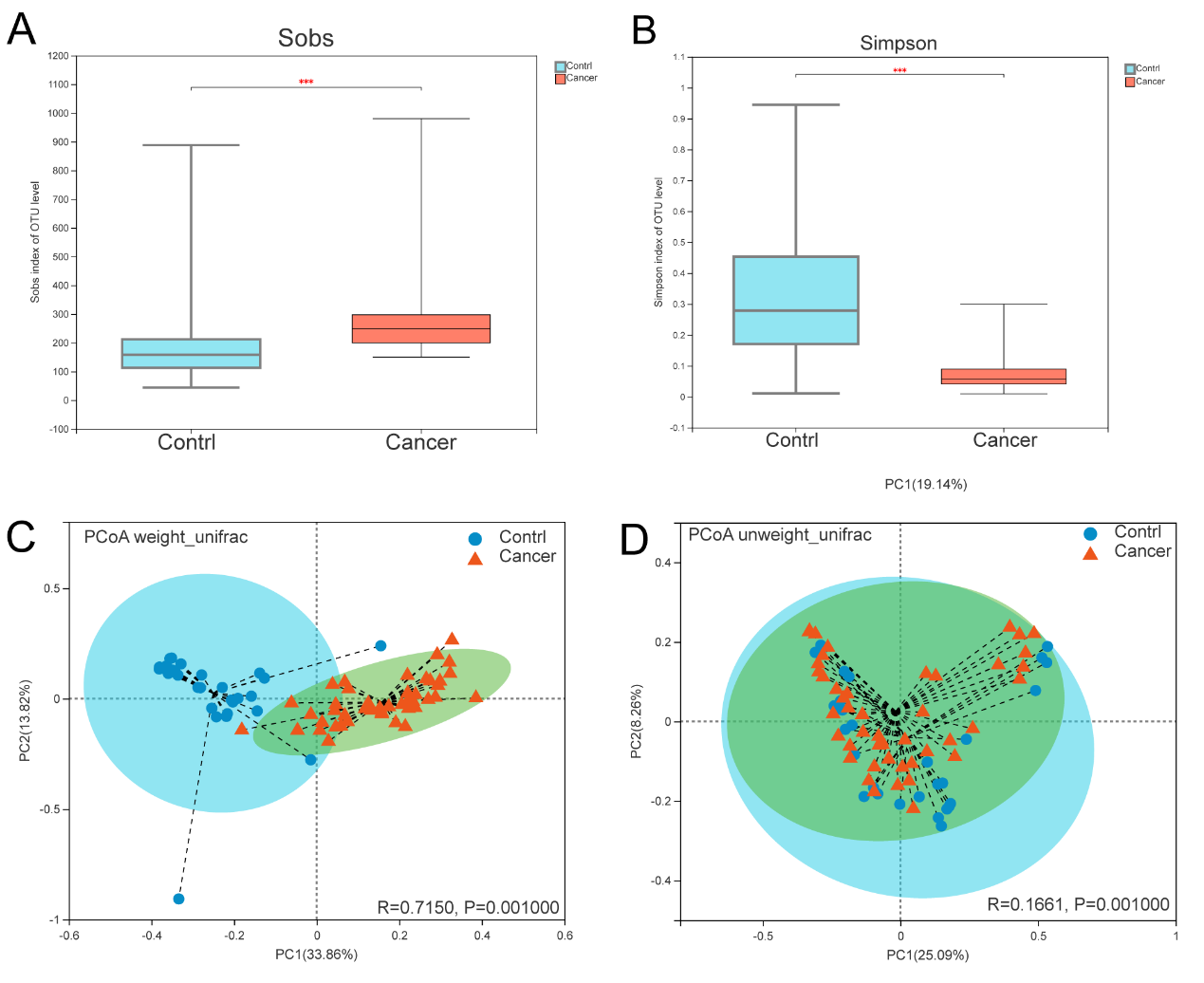


Figure S1 α and β diversity for the microbial community of samples from the lung cancer patients and control group. α-diversity analysis with different parameters. Sobs (A) and Simpson index (B). β-diversity analysis with Principal coordinate analysis (PCoA) plots of the weighted UniFrac(C), and unweighted UniFrac(D) distance matrix.


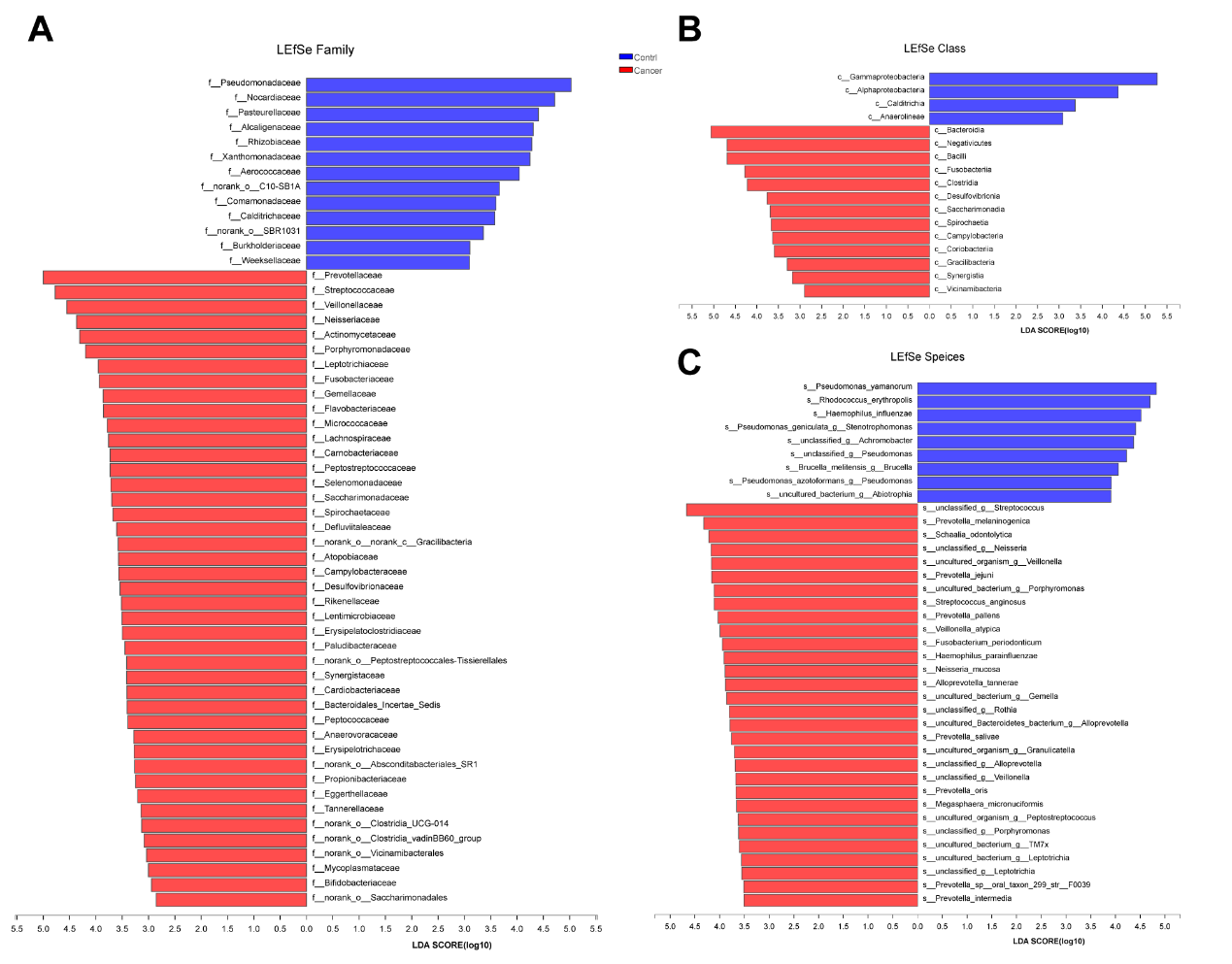


Figure S2 (A) LDA scores showed significant bacterial differences within groups at the family level. (B) LDA scores showed significant bacterial differences within groups at the class level. (C) LDA scores showed significant bacterial differences within groups at the species level. Blue indicates taxa enriched in control patients, and red indicates taxa enriched in NSCLC cancer.


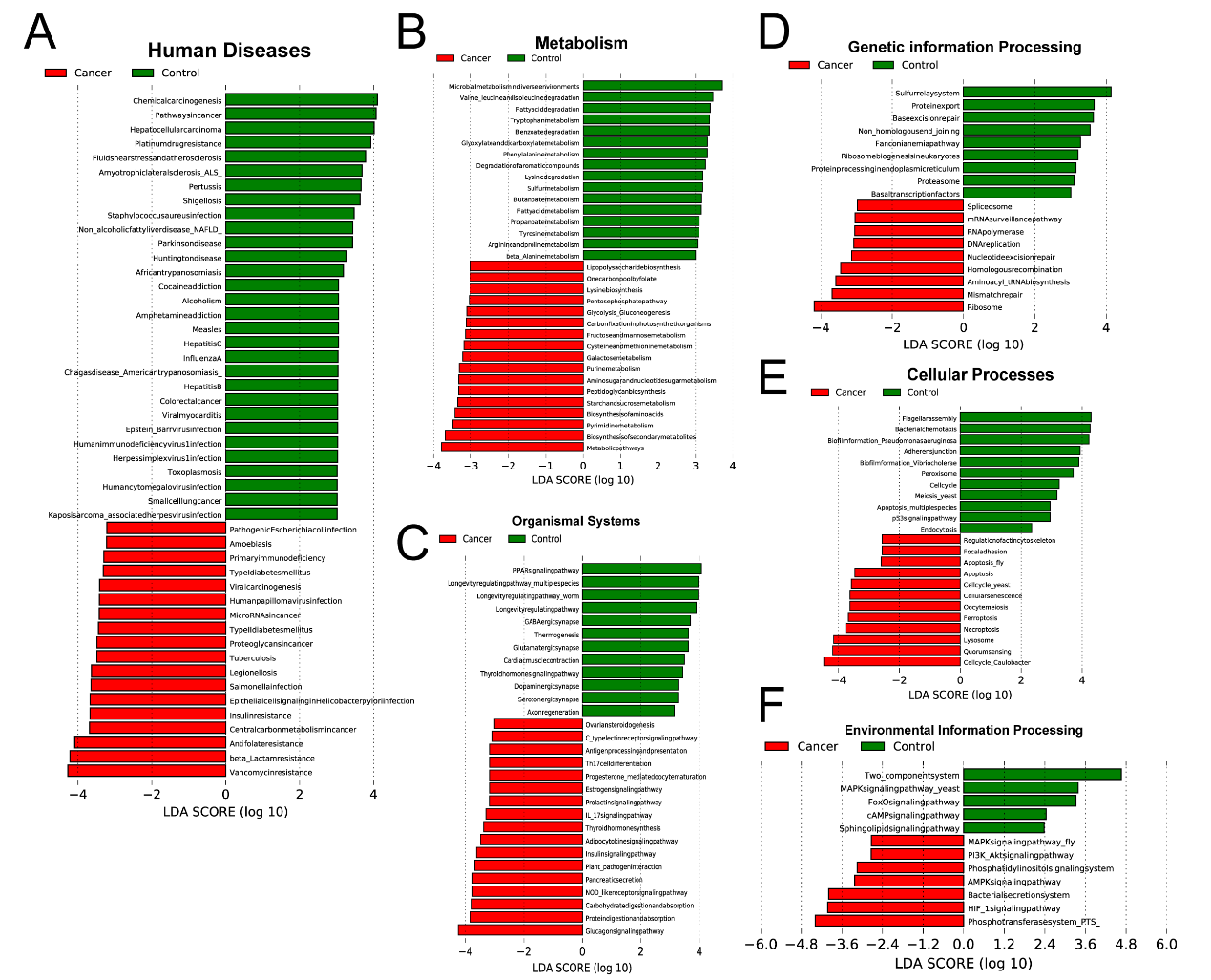


Figure S3. Prediction of the lung microbiota gene function using PICRUSt2. Human Diseases(A), Metabolism(B), Organismal Systems(C), Genetic information processing(D), meeting an LDA score >3.0 are shown. Cellular process (E), Environmental information processing (F) meeting an LDA score >2.0 are shown.Red and green histograms represent cancer and control respectively.
