# Supplementary material for: Alterations of lung microbiota in patients with non-small cell lung cancer
Source: Bioengineered. 2022 Mar 7;13(3):6665–77. doi: 10.1080/21655979.2022.2045843 (PMC8973753; doi:10.1080/21655979.2022.2045843)
Supplement: Supplemental Material [file KBIE_A_2045843_SM6988.zip › 补充结果/Table S1.docx]

| **Table S1-sequencing data statis** | | | | | | |
| --- | --- | --- | --- | --- | --- | --- |
| Group | Sample\Info | Seq_num | Base_num | Mean_length | Min_length | Max_length |
| Normal | A1 | 64329 | 26923596 | 418.529683 | 207 | 510 |
| Normal | A10 | 35465 | 14762639 | 416.259382 | 312 | 432 |
| Normal | A11 | 57347 | 24515250 | 427.489668 | 253 | 452 |
| Normal | A28 | 34924 | 14921276 | 427.249914 | 206 | 536 |
| Normal | A3 | 62517 | 26750851 | 427.897228 | 322 | 478 |
| Normal | A30 | 35495 | 15142717 | 426.615495 | 244 | 478 |
| Normal | A31 | 55525 | 23002803 | 414.278307 | 204 | 529 |
| Normal | A36 | 41980 | 17999859 | 428.772249 | 325 | 449 |
| Normal | A4 | 50895 | 21317254 | 418.847706 | 250 | 452 |
| Normal | A43 | 46846 | 19567958 | 417.708193 | 200 | 524 |
| Normal | A5 | 87843 | 37084463 | 422.167538 | 207 | 507 |
| Normal | A50 | 33750 | 14152314 | 419.327822 | 200 | 518 |
| Normal | A52 | 35545 | 14900732 | 419.20754 | 200 | 534 |
| Normal | A55 | 57057 | 21221607 | 371.936958 | 200 | 527 |
| Normal | A56 | 45579 | 18868194 | 413.966827 | 200 | 499 |
| Normal | A57 | 68889 | 29086833 | 422.22754 | 200 | 507 |
| Normal | A60 | 70132 | 29617918 | 422.316746 | 200 | 497 |
| Normal | A61 | 68539 | 29098480 | 424.553612 | 200 | 479 |
| Normal | A63 | 78399 | 33299631 | 424.745609 | 201 | 534 |
| Normal | A64 | 65279 | 27903798 | 427.454434 | 200 | 485 |
| Normal | A65 | 66480 | 28137897 | 423.253565 | 200 | 513 |
| Normal | A66 | 76393 | 32701391 | 428.067899 | 207 | 510 |
| Normal | A67 | 70041 | 29362366 | 419.21683 | 200 | 464 |
| Normal | A7 | 47844 | 20067961 | 419.445719 | 202 | 521 |
| Normal | A70 | 60989 | 26146462 | 428.707833 | 206 | 484 |
| Normal | A77 | 70912 | 29866385 | 421.17533 | 201 | 485 |
| Normal | A78 | 66919 | 28342123 | 423.528789 | 200 | 478 |
| Normal | A80 | 69364 | 29316126 | 422.641803 | 200 | 440 |
| Normal | A9 | 60677 | 25497650 | 420.219358 | 259 | 493 |
| Cancer | A12 | 59985 | 25306609 | 421.882287 | 213 | 514 |
| Cancer | A13 | 43417 | 18367391 | 423.046065 | 207 | 469 |
| Cancer | A17 | 70460 | 29962673 | 425.243727 | 231 | 521 |
| Cancer | A18 | 36645 | 15543180 | 424.155546 | 257 | 469 |
| Cancer | A19 | 59608 | 25012095 | 419.6097 | 244 | 462 |
| Cancer | A2 | 52688 | 22056858 | 418.631529 | 400 | 431 |
| Cancer | A20 | 46872 | 19775080 | 421.895375 | 241 | 514 |
| Cancer | A21 | 39374 | 16693606 | 423.975364 | 202 | 503 |
| Cancer | A22 | 58752 | 24920922 | 424.171467 | 206 | 518 |
| Cancer | A23 | 63795 | 26754121 | 419.376456 | 201 | 513 |
| Cancer | A24 | 47276 | 20132326 | 425.846645 | 202 | 459 |
| Cancer | A25 | 32842 | 13938669 | 424.415961 | 250 | 507 |
| Cancer | A26 | 42569 | 17892005 | 420.305974 | 250 | 458 |
| Cancer | A27 | 43485 | 18414584 | 423.469794 | 231 | 531 |
| Cancer | A32 | 33976 | 14423488 | 424.519896 | 200 | 452 |
| Cancer | A33 | 61335 | 25943704 | 422.98368 | 202 | 507 |
| Cancer | A35 | 53122 | 22572806 | 424.923873 | 200 | 505 |
| Cancer | A37 | 38205 | 15984249 | 418.381076 | 204 | 507 |
| Cancer | A38 | 62409 | 26487133 | 424.412072 | 245 | 510 |
| Cancer | A39 | 44020 | 18624084 | 423.082326 | 207 | 463 |
| Cancer | A41 | 49121 | 20869240 | 424.853729 | 200 | 534 |
| Cancer | A42 | 44931 | 19039698 | 423.754156 | 200 | 540 |
| Cancer | A44 | 48980 | 20582787 | 420.228399 | 207 | 517 |
| Cancer | A45 | 60255 | 25060550 | 415.908223 | 200 | 528 |
| Cancer | A46 | 42310 | 18036121 | 426.285063 | 202 | 517 |
| Cancer | A47 | 32428 | 13621014 | 420.03867 | 200 | 534 |
| Cancer | A48 | 35256 | 14925333 | 423.341644 | 200 | 477 |
| Cancer | A49 | 46423 | 19684314 | 424.020722 | 202 | 499 |
| Cancer | A51 | 41816 | 17647078 | 422.017362 | 200 | 507 |
| Cancer | A53 | 30515 | 12799677 | 419.455252 | 200 | 539 |
| Cancer | A54 | 50638 | 21264779 | 419.937182 | 200 | 517 |
| Cancer | A58 | 81784 | 34639623 | 423.55012 | 245 | 510 |
| Cancer | A59 | 73877 | 30999695 | 419.612261 | 231 | 462 |
| Cancer | A6 | 126467 | 53436097 | 422.529964 | 268 | 507 |
| Cancer | A62 | 77011 | 32608113 | 423.421498 | 241 | 507 |
| Cancer | A69 | 61330 | 26085152 | 425.324507 | 200 | 528 |
| Cancer | A71 | 85475 | 36103213 | 422.383305 | 224 | 486 |
| Cancer | A72 | 69752 | 29585469 | 424.152268 | 253 | 495 |
| Cancer | A73 | 80563 | 34092341 | 423.17616 | 206 | 530 |
| Cancer | A74 | 70779 | 30092767 | 425.16519 | 239 | 487 |
| Cancer | A75 | 77774 | 32986109 | 424.127716 | 200 | 452 |
| Cancer | A76 | 80872 | 33116145 | 409.488389 | 234 | 531 |
| Cancer | A79 | 60753 | 25841485 | 425.353234 | 200 | 522 |
| Cancer | A8 | 78029 | 33011412 | 423.065937 | 207 | 507 |
| Cancer | C2 | 40695 | 17148414 | 421.388721 | 250 | 489 |
| Cancer | C3 | 47895 | 20208150 | 421.926088 | 250 | 522 |
