# Supplementary material for: Alterations of lung microbiota in patients with non-small cell lung cancer
Source: Bioengineered. 2022 Mar 7;13(3):6665–77. doi: 10.1080/21655979.2022.2045843 (PMC8973753; doi:10.1080/21655979.2022.2045843)
Supplement: Supplemental Material [file KBIE_A_2045843_SM6988.zip › 补充结果/Table S2.docx]

| Table S2. The unique microbiota of phylotypes in lung cancer group and healthy group | | |
| --- | --- | --- |
| Phylotype | Group (number) |  |
| OUT | Control only(13) | OTU1568 OTU4755 OTU5099 OTU4594 OTU1717 OTU6524 OTU6443 OTU1503 OTU5110 OTU2129 OTU4807 OTU4481 OTU1339 |
|  | Lung cancer only(66) | OTU5559 OTU5313 OTU333 OTU4920 OTU1923 OTU346 OTU6001 OTU2513 OTU6098 OTU6093 OTU6097 OTU6781 OTU5146 OTU377 OTU106 OTU5187 OTU5717 OTU5714 OTU5844 OTU5841 OTU33 OTU4918 OTU5327 OTU6301 OTU6306 OTU402 OTU407 OTU6708 OTU1268 OTU5531 OTU5383 OTU5988 OTU6634 OTU6448 OTU692 OTU6118 OTU5833 OTU5838 OTU6244 OTU6061 OTU6065 OTU2539 OTU2535 OTU5185 OTU5309 OTU5732 OTU5307 OTU1860 OTU5426 OTU4882 OTU5998 OTU221 OTU731 OTU359 OTU6102 OTU5280 OTU6079 OTU5596 OTU5896 OTU5953 OTU5293 OTU5128 OTU5512 OTU6659 OTU5706 OTU6045 |
| Genus | Control only(7) | g__Elizabethkingia g__norank_f__norank_o__Subgroup_7 g__Dickeya g__Filimonas g__OLB12 g__Virgibacillus g__norank_f__norank_o__Bacteroidetes_VC2.1_Bac22 |
|  | Lung cancer only(26) | g__norank_f__norank_o__norank_c__norank_p__Firmicutes g__Prevotellaceae_UCG-001 g__norank_f__norank_o__Actinomarinales g__Vicinamibacter g__Planifilum g__Helicobacter g__norank_f__Dysgonomonadaceae g__norank_f__Microscillaceae g__Curvibacter g__Allobaculum g__Kingella g__Christensenellaceae_R-7_group g__Sedimentibacter g__Dorea g__Alysiella g__Lachnospiraceae_NK4A136_group g__norank_f__Eubacterium_coprostanoligenes_group g__norank_f__Bacteroidales_UCG-001 g__Halocella g__Amnipila g__Acetitomaculum g__Desulfovibrio g__Herbinix g__Coriobacteriaceae_UCG-002 g__norank_f__Desulfovibrionaceae g__Caldicoprobacter |
| Species | Control only(7) | s__Virgibacillus_siamensis s__uncultured_bacterium_g__OLB12 s__metagenome_g__norank_f__norank_o__Bacteroidetes_VC2.1_Bac22 s__Filimonas_sp. s__Oscillatoriales_cyanobacterium_HF1 s__Dickeya_zeae s__Elizabethkingia_miricola s__uncultured_Acidobacterium_sp._g__norank_f__norank_o__Subgroup_7 |
|  | Lung cancer only(47) | s__Planifilum_composti s__Helicobacter_ganmani s__uncultured_bacterium_g__Christensenellaceae_R-7_group s__Veillonellaceae_bacterium_oral_taxon_145 s__uncultured_bacterium_g__Kingella s__uncultured_bacterium_g__Coriobacteriaceae_UCG-002 s__Dialister_micraerophilus s__uncultured_bacterium_g__Lachnospiraceae_NK3A20_group s__uncultured_prokaryote_g__norank_f__norank_o__norank_c__norank s__uncultured_bacterium_g__Bacteroides s__uncultured_Veillonellaceae_bacterium_g__Phascolarctobacterium s__uncultured_bacterium_g__norank_f__Desulfovibrionaceae s__gut_metagenome_g__norank_f__Eubacterium_coprostanoligenes_group s__unclassified_g__norank_f__Muribaculaceae s__uncultured_rumen_bacterium_g__Acetitomaculum s__uncultured_bacterium_g__Alysiella s__uncultured_bacterium_g__norank_f__norank_o__Clostridia_UCG-014 s__Prevotella_oralis s__uncultured_soil_bacterium_g__norank_f__Dysgonomonadaceae s__uncultured_bacterium_g__Allobaculum s__uncultured_bacterium_g__Treponema s__uncultured_Acidobacteriaceae_bacterium_g__norank_f__Vicinamibacteraceae s__uncultured_Actinomyces_sp._g__Actinomyces s__Herbinix_luporum s__uncultured_bacterium_g__Prevotellaceae_UCG-001 s__Lachnospiraceae_bacterium_28-4 s__uncultured_compost_bacterium_g__norank_f__norank_o__Actinomarinales s__Mitsuokella_sp._oral_taxon_131_str._W9106 s__unclassified_g__Curvibacter s__uncultured_bacterium_g__Olsenella s__uncultured_rumen_bacterium_g__NK4A214_group s__mouse_gut_metagenome_g__norank_f__Muribaculaceae s__uncultured_bacterium_g__Desulfovibrio s__uncultured_organism_g__Amnipila s__uncultured_bacterium_g__norank_f__norank_o__norank_c__Gitt-GS-136 s__metagenome_g__Desulfovibrio s__uncultured_bacterium_g__Halocella s__anaerobic_digester_metagenome_g__Caldicoprobacter s__unclassified_g__norank_f__Microscillaceae s__uncultured_methanogenic_archaeon_g__Veillonella s__uncultured_bacterium_g__norank_f__Bacteroidales_UCG-001 s__unclassified_g__Shuttleworthia s__Dorea_formicigenerans_ATCC_27755 s__uncultured_bacterium_g__Sedimentibacter s__uncultured_organism_g__norank_f__Muribaculaceae s__uncultured_Clostridiales_bacterium_g__Lachnospiraceae_NK4A136_group s__uncultured_bacterium_g__Vicinamibacter |
| Phylum | Control only(0) | -- |
|  | Lung cancer only(1) | p__Halanaerobiaeota |
