# Supplementary figures and images for: Alterations of lung microbiota in patients with non-small cell lung cancer
Source: Bioengineered. 2022 Mar 7;13(3):6665–77. doi: 10.1080/21655979.2022.2045843 (PMC8973753; doi:10.1080/21655979.2022.2045843)

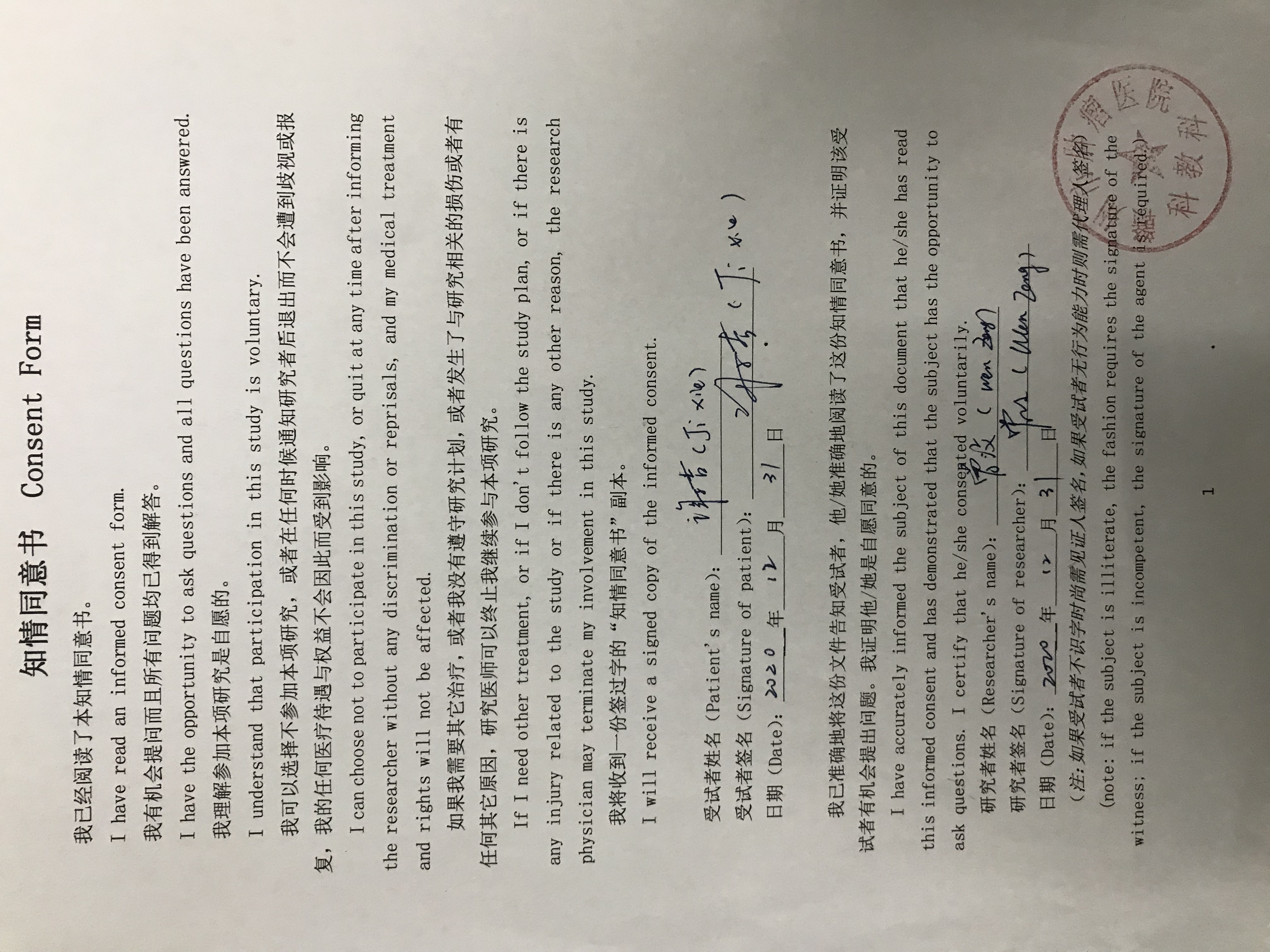

Supplement: Supplemental Material [file KBIE_A_2045843_SM6988.zip › 补充结果/Consent Form.jpg]

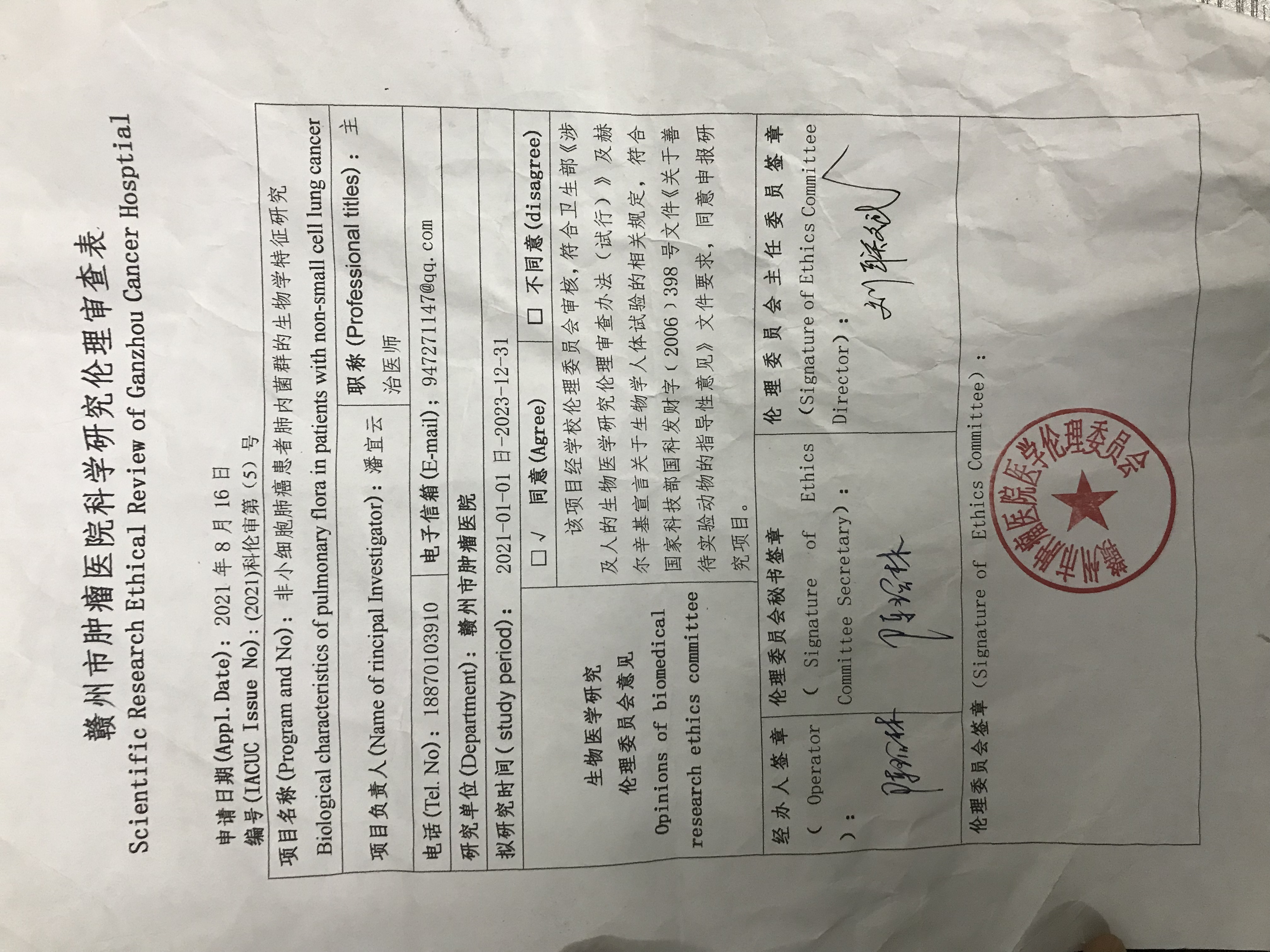

Supplement: Supplemental Material [file KBIE_A_2045843_SM6988.zip › 补充结果/Ethical review.jpg]

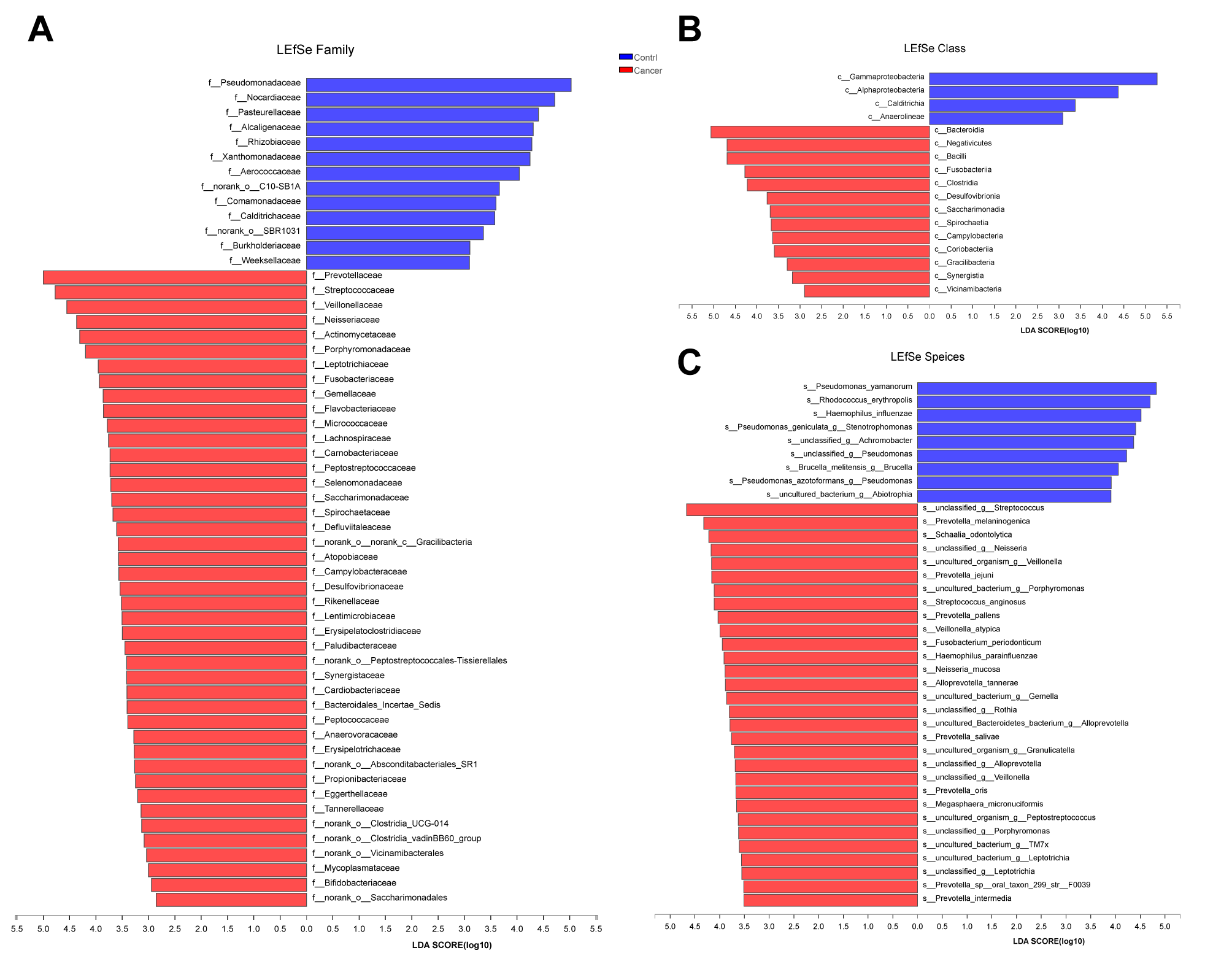

Supplement: Supplemental Material [file KBIE_A_2045843_SM6988.zip › 补充结果/Figure S2.tif]

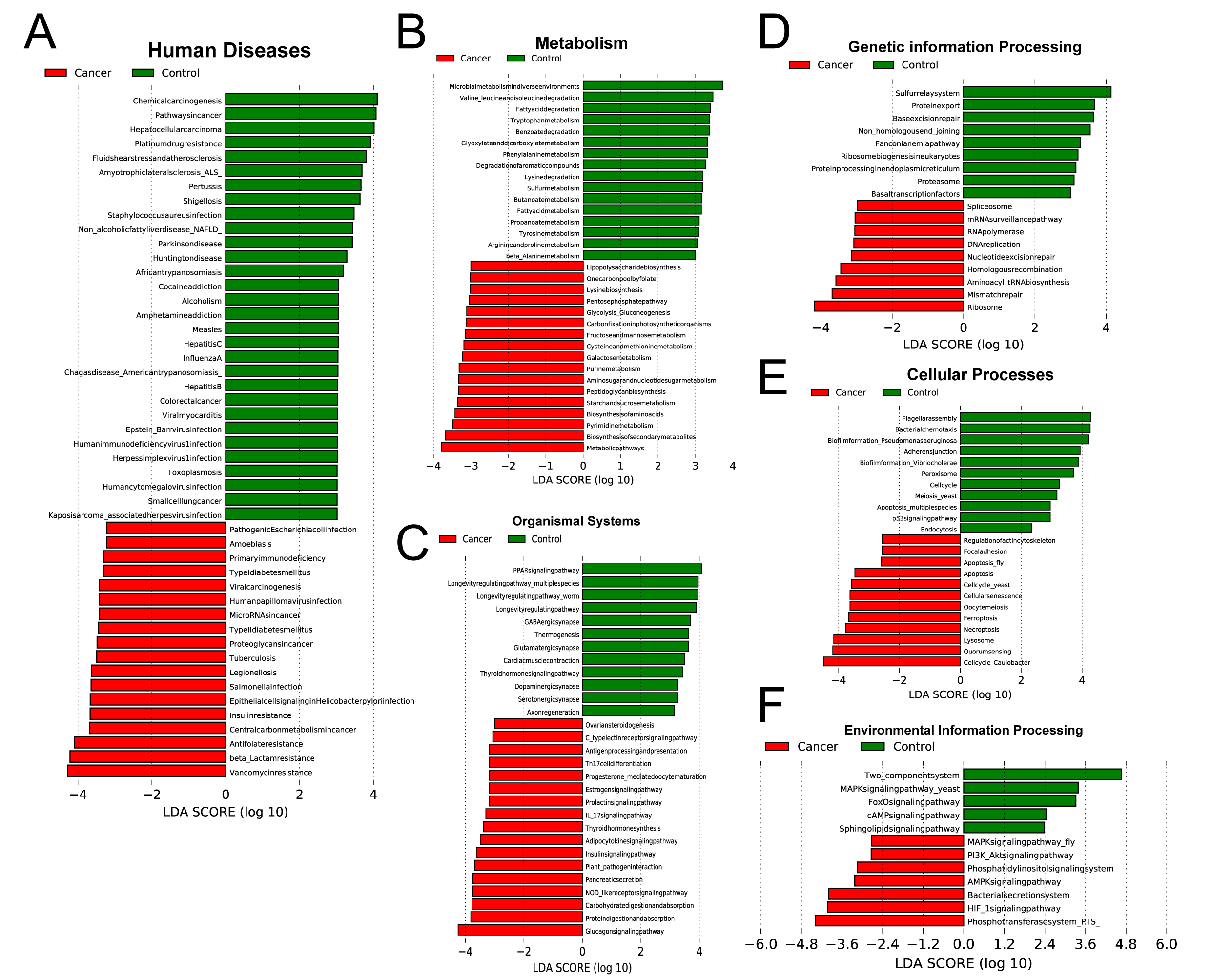

Supplement: Supplemental Material [file KBIE_A_2045843_SM6988.zip › 补充结果/Figure S3.tif]

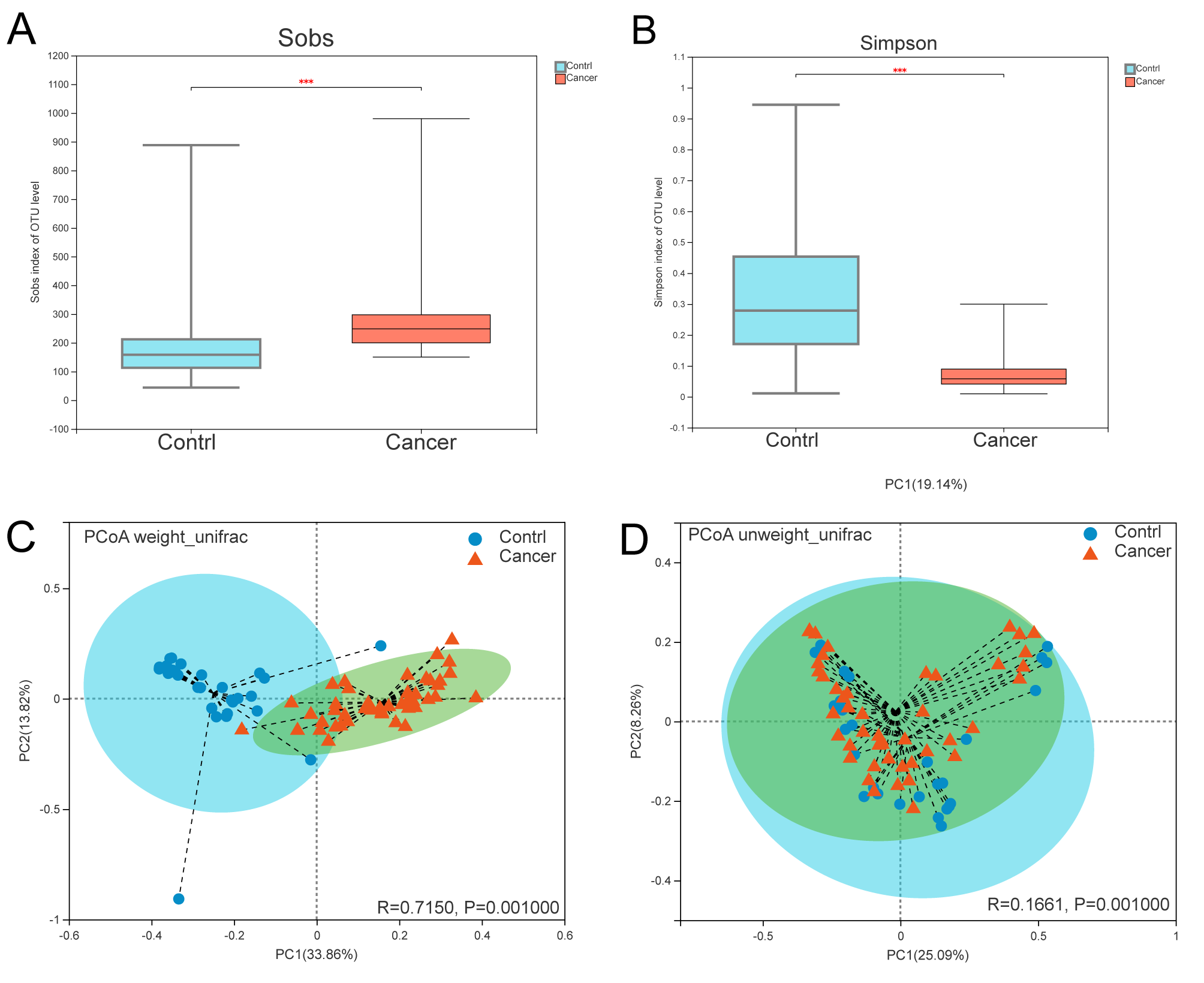

Supplement: Supplemental Material [file KBIE_A_2045843_SM6988.zip › 补充结果/Fiigure S1.tif]
